# Regional profiling reveals a distinct glioblastoma infiltrative margin proteome

**DOI:** 10.1038/s41598-025-09228-z

**Published:** 2025-07-05

**Authors:** Artur Kocon, Stuart J. Smith, Benito Morentin, Luis F. Callado, Wayne Carter, Ruman Rahman

**Affiliations:** 1 Clinical Toxicology Research Group, School of Medicine, Royal Derby Hospital Centre, University of Nottingham, Uttoxeter Road, Derby, DE22 3DT UK; 2 School of Medicine, Biodiscovery Institute, University of Nottingham, University Park, Nottingham, NG7 2RD UK; 3 Section of Forensic Pathology, Basque Institute of Legal Medicine, 48001 Bilbao, Spain; 4 BioBizkaia Health Research Institute, 48903 Barakaldo, Bizkaia Spain; 5 Department of Pharmacology, University of the Basque Country-UPV/EHU, 48940 Leioa, Bizkaia Spain; 6 Centro de Investigación Biomédica en Red de Salud Mental (CIBERSAM), Madrid, Spain

**Keywords:** Glioblastoma, 2D-PAGE, Proteomics, Tandem mass spectrometry, CNS cancer, Biomarkers, Proteomics

## Abstract

Isocitrate dehydrogenase wild-type glioblastoma, a malignant brain tumour of glial origin, confers a poor prognosis with a median survival of 12 to 16 months from diagnosis. Glioblastomas are aggressive tumours that rapidly proliferate and diffusely infiltrate surrounding brain tissue. Current multimodal standard treatment is typically ineffective and despite gross total surgical resection, tumours recur with more aggressive sub-clonal populations of malignant cells. A defining characteristic of glioblastoma is its highly heterogeneous nature and acquirement of somatic mutations advantageous to tumour growth and suppression of apoptotic pathways. Pathogenesis of malignant brain tumours as well as its mode of transformation to a more aggressive subtype is still largely unknown. Although genomic studies have elucidated a plethora of genetic markers associated with glioblastoma subtypes, only a few have been utilised in a clinical setting. One of the emerging approaches to studying glioblastomas is by investigating how an active proteome contributes to its aggressive nature. Furthermore, through activation of specific pathways via post-translational modifications of proteins such as phosphorylation, glioblastomas create an intricate network of signalling pathways which favour tumour growth and proliferation. Here, we investigated the feasibility of diverse methodological approaches to describe abnormal protein signalling across distinct intra-tumour regions of primary glioblastoma tissue, including proliferative core, peripheral rim, and invasive margin. Whilst we observe a broadly comparable proteome relative to the human non-diseased brain, we identify cytoplasmic proteins α-trypsin, actin, apolipoprotein A1 and transthyretin which may putatively be associated with the GBM infiltrative tumour margin.

**Supplementary Information:**

The online version contains supplementary material available at 10.1038/s41598-025-09228-z.

## Introduction

The discovery of biological markers (biomarkers) of disease has had a tremendous impact on the classification and treatment of disease. This has led to a new era where the characterisation of the active human proteome and biomarker discovery has been established as one of the principal challenges in our understanding of health and disease^1^. Proteomics refers to the large-scale characterisation of proteins encoded by the genome, whereas the proteome can be defined as the complete protein content of a cell or a tissue. It serves as a complementary system to genomics to further define the physiological outcome of genes and provide a more informative medium for cellular function^2^. Similarly to the Human Genome Project, several projects have been established in order to catalogue and map the human proteome such as the Human Proteome Project, Human Protein Atlas and ProteomeXchange consortium, which facilitates the sharing of proteomic data^3–5^. Compiling an extensive proteome map could enable and initiate a more efficient path for proteomic research and understanding of various cancers and other diseases. Ideally, through utilizing both, genomic and proteomic information, a more comprehensive picture of human physiology as well as pathology can be achieved to offer more personalised medicine.

As an exemplar of treatment-intractable human disease for which proteomic approaches have yet to yield clinically-actionable signatures, we focused on isocitrate dehydrogenase wild-type glioblastoma (GBM). Classified as World Health Organisation grade 4 astrocytomas, GBMs are neuroepithelial tumours that are highly heterogeneous and characterised by extensive sub-clonal divergence. The prognosis of patients diagnosed with GBM is poor, as the median survival is 12–16 months from diagnosis, despite neurosurgical resection of the entire bulk tumour and adjuvant treatment with radio- and temozolomide chemo-therapy^6^. True complete surgical resection is not possible, due to the residual disease widely infiltrating neighbouring brain parenchyma. As a result, recurrence is inevitable, with tumour relapse typically occurring within 2 cm of the primary tumour infiltrative margin^7^. The evolutionary dynamics of GBMs are directly associated with versatility to adapt to changing microenvironments, facilitating rapid growth and diffuse infiltration into brain parenchyma^8–10^. Acquisition of somatic mutations with time and across various intra-tumour regions makes it difficult to define a single biomarker specific to GBM. Identification of specific therapeutic targets is often more viable, but due to GBMs’ high heterogeneity, sub-clonal populations with advantageous mutations conferred by chemotherapy or radiotherapy, emerge and predominate the tumour with subsequent greater resistance to therapeutic agents.

Microarray profiling and next-generation sequencing have provided a further delineation of GBM subtypes expanding the classical histology/grade-based glioma diagnosis^11^. Several studies have reported altered protein regulation between different grades of gliomas using proteomics-based technologies. Ren Tong et al.^12^ conducted an iTRAQ-based quantitative proteomics study to compare the proteomic profile of different grades of astrocytoma and reported that several proteins such as metalloproteinase 9 and metalloproteinase inhibitor 1 were upregulated in GBMs. Other proteins including fibulin 2 and fibulin 5 were downregulated in higher grades which was associated with advanced disease. Integrated proteo-genomic studies have also reported upregulation of gene sets including transforming growth factor-β1 correlated with shorter overall survival^13^, and recently, a longitudinal analysis revealed a proteomic switch from a highly proliferative cellular stage at diagnosis, to activation of neuronal transition and synaptogenic pathways in recurrent tumours associated with post-translational activation of the wingless-related integration site/ planar cell polarity signalling pathway and BRAF protein kinase^14^. Whilst these reports suggest that protein profiling and proteomics are valuable genome-wide platforms to study differential regulation of GBM pathophysiology, the proteome which manifests in GBM residual disease remains poorly understood. We have shown that RNA-sequencing-based transcriptomic profiles of the GBM infiltrative margin (as a proxy for residual disease), not only exhibit significantly divergent gene expression patterns to the resected tumour core but that this expression profile is more similar a priori to the eventual GBM recurrence^15,16^. Therefore, to facilitate future intra-tumour investigations of the GBM proteome, we here establish the methodological feasibility of applying diverse protein profiling techniques to surgical biopsy tissue.

## Results

### Multiplex immunoassay reveals CREB and Akt upregulation in the GBM invasive margin

Cytosolic and nuclear proteins were isolated from primary GBM biopsies derived from four consented patients who had undergone resection surgery at Queen’s Medical Centre, Nottingham University Hospitals, Nottingham. Tissue was obtained from five intra-tumour regions per patient, including tumour core, peripheral (non-invasive) rim, and 5-aminolevulinic (5ALA)-based fluorescing invasive margin (Table 1 and Supplementary Table 1). As the GBM invasive margin resides in closest proximity to residual disease spared by surgery, we sought to identify unique or significantly altered protein signatures within this clinically relevant tumour region.

**Table 1:** GBM sample coding and demographics.

Tissue ID	Intra-tumour region	Tumour site	Gender	Age
GBM 38.1	Superficial fluorescence (Tumour Rim)	Right frontal hemisphere	Male	69
GBM 38.2	Central core			
GBM 38.3	Central core 2			
GBM 38.4	Deep lateral fluorescence (Tumour Rim)			
GBM 38.5	Invasive margin			
GBM 39.1	Superficial fluorescence (Tumour Rim)	Right frontal hemisphere	Female	58
GBM 39.2	Central core			
GBM 39.3	Posterior core			
GBM 39.4	Anterior enhancement (Tumour Rim)			
GBM 39.5	Invasive margin			
GBM 40.1	Superior enhancement (Tumour Rim)	Right frontal hemisphere	Male	44
GBM 40.2	Central core			
GBM 40.3	Anterior enhancement (Tumour Rim)			
GBM 40.4	Posterior enhancement (Tumour Rim)			
GBM 40.5	Invasive margin			
GBM 58.1	Superficial enhancement (Tumour Rim)	Left frontal hemisphere	Male	78
GBM 58.2	Necrotic core			
GBM 58.3	Posterior rim (Tumour Rim)			
GBM 58.4	Anterior rim (Tumour Rim)			
GBM 58.5	Invasive margin			

GBM samples used in the study with sample ID and distinct tumour regions divided into tumour rim, tumour core and invasive margin. The distinct areas have been outlined by the surgeon with 5ALA-assisted surgery and specified areas have been separated.

We first conducted a quantitative immunoassay using a Milliplex^®^ multiplex assay to identify total and phosphorylated cytosolic proteins from canonical biochemical signalling pathways associated with cell migration, cell cycle control, transcription, cell survival and proliferation. Three distinct assays were used to investigate a panel of phosphorylated proteins (9-Plex multi-pathway) and phosphorylated mTOR and JNK specifically (Supplementary Table 2). Four GBM tumours (patients 38, 39, 40 and 58) and three distinct regions from the tumour core, tumour rim and invasive margin, were investigated relative to tissue from human prefrontal cortex non-diseased controls (Table 1).

**Fig. 1:**
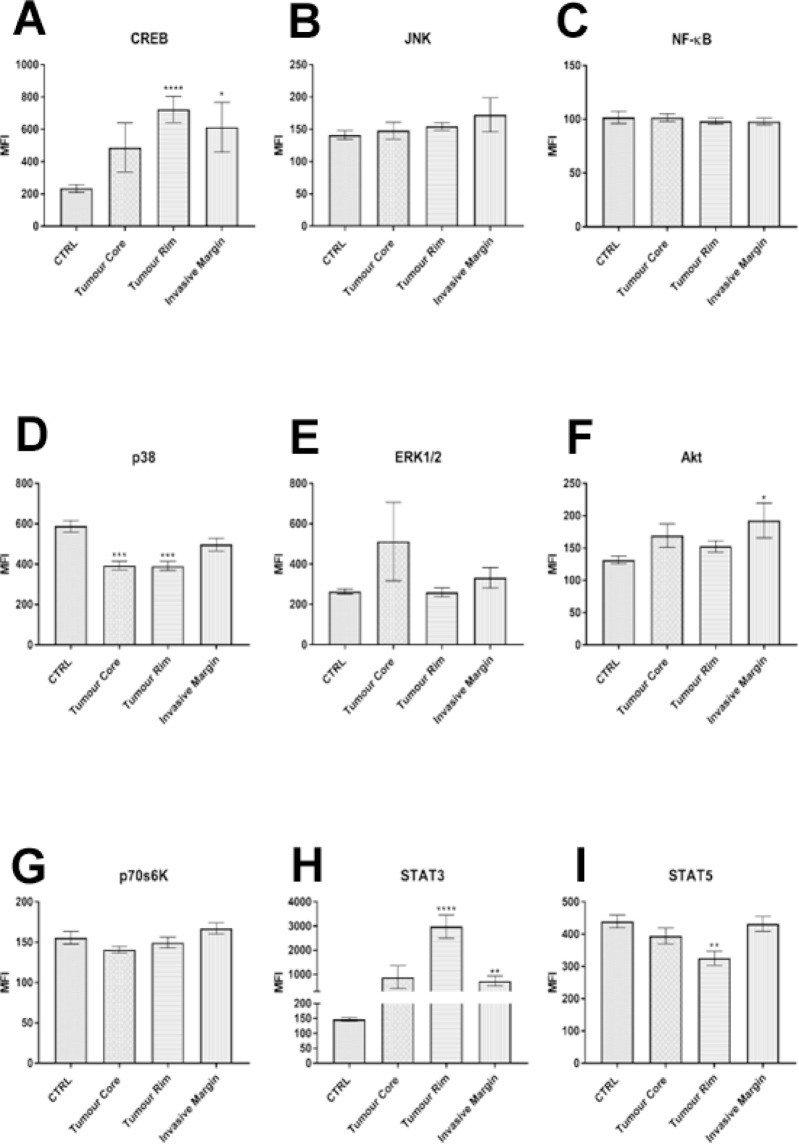
Expression of cytosolic phosphorylated proteins assessed through Milliplex MAP technology. Expression is plotted as the Median Fluorescent Intensity (MFI) of cytosolic proteins in intra-tumour GBM regions and prefrontal cortex controls (CTRL). Data was analysed by non-parametric one-way ANOVA (Kruskal–Wallis test) and statistical significance set at *P < 0.05; **P < 0.01; ***P < 0.001. Mean ± SEM plotted.

Cytosolic levels of CREB (*p* = 0.0331), and STAT3 (*p* = 0.0056) were significantly upregulated in the GBM invasive margin (mean across all four patients) relative to non-diseased brain controls. Akt (*p* = 0.0366) was also uniquely elevated in the invasive margin, suggesting a putative role for CREB-mediated transcriptional regulation and Akt-mediated cellular survival in promoting an invasive phenotype. None of the phosphorylated cytosolic proteins that were examined were upregulated in the GBM tumour core relative to the non-diseased brains; however, p38 was significantly downregulated (*p* = 0.0008). CREB (*p* < 0.0001) and STAT3 (*p* < 0.0001) phosphorylated cytosolic proteins were significantly upregulated, and p38 (*p* = 0.0001) and STAT5 (*p* = 0.0022) significantly downregulated in the GBM peripheral rim relative to the non-diseased brains (Fig. 1 and Table 2).

**Table 2:** Multiple comparisons of Median Fluorescent Intensity (MFI) from Milliplex Map 9Plex analysis between GBM intra-tumour regions and prefrontal cortex control (CTRL).

CREB	CTRL vs. Tumour Core	ns	0.5872
	CTRL vs. Tumour Rim	****	< 0.0001
	CTRL vs. Invasive Margin	*	0.0331
JNK	CTRL vs. Tumour Core	ns	> 0.9999
	CTRL vs. Tumour Rim	ns	0.6849
	CTRL vs. Invasive Margin	ns	> 0.9999
NF-κB	CTRL vs. Tumour Core	ns	> 0.9999
	CTRL vs. Tumour Rim	ns	> 0.9999
	CTRL vs. Invasive Margin	ns	> 0.9999
p38	CTRL vs. Tumour Core	***	0.0008
	CTRL vs. Tumour Rim	***	0.0001
	CTRL vs. Invasive Margin	ns	0.5151
ERK1/2	CTRL vs. Tumour Core	ns	0.4996
	CTRL vs. Tumour Rim	ns	0.373
	CTRL vs. Invasive Margin	ns	> 0.9999
Akt	CTRL vs. Tumour Core	ns	0.3064
	CTRL vs. Tumour Rim	ns	0.3697
	CTRL vs. Invasive Margin	*	0.0366
p70 S6K	CTRL vs. Tumour Core	ns	0.3807
	CTRL vs. Tumour Rim	ns	> 0.9999
	CTRL vs. Invasive Margin	ns	0.5826
STAT3	CTRL vs. Tumour Core	ns	0.1201
	CTRL vs. Tumour Rim	****	< 0.0001
	CTRL vs. Invasive Margin	**	0.0056
STAT5	CTRL vs. Tumour Core	ns	0.8033
	CTRL vs. Tumour Rim	**	0.0022
	CTRL vs. Invasive Margin	ns	> 0.9999

Data was analysed by non-parametric one-way ANOVA (Kruskal–Wallis test) and statistical significance set at *P < 0.05; **P < 0.01; ***P < 0.001;****P < 0.0001.

**Fig. 2:**
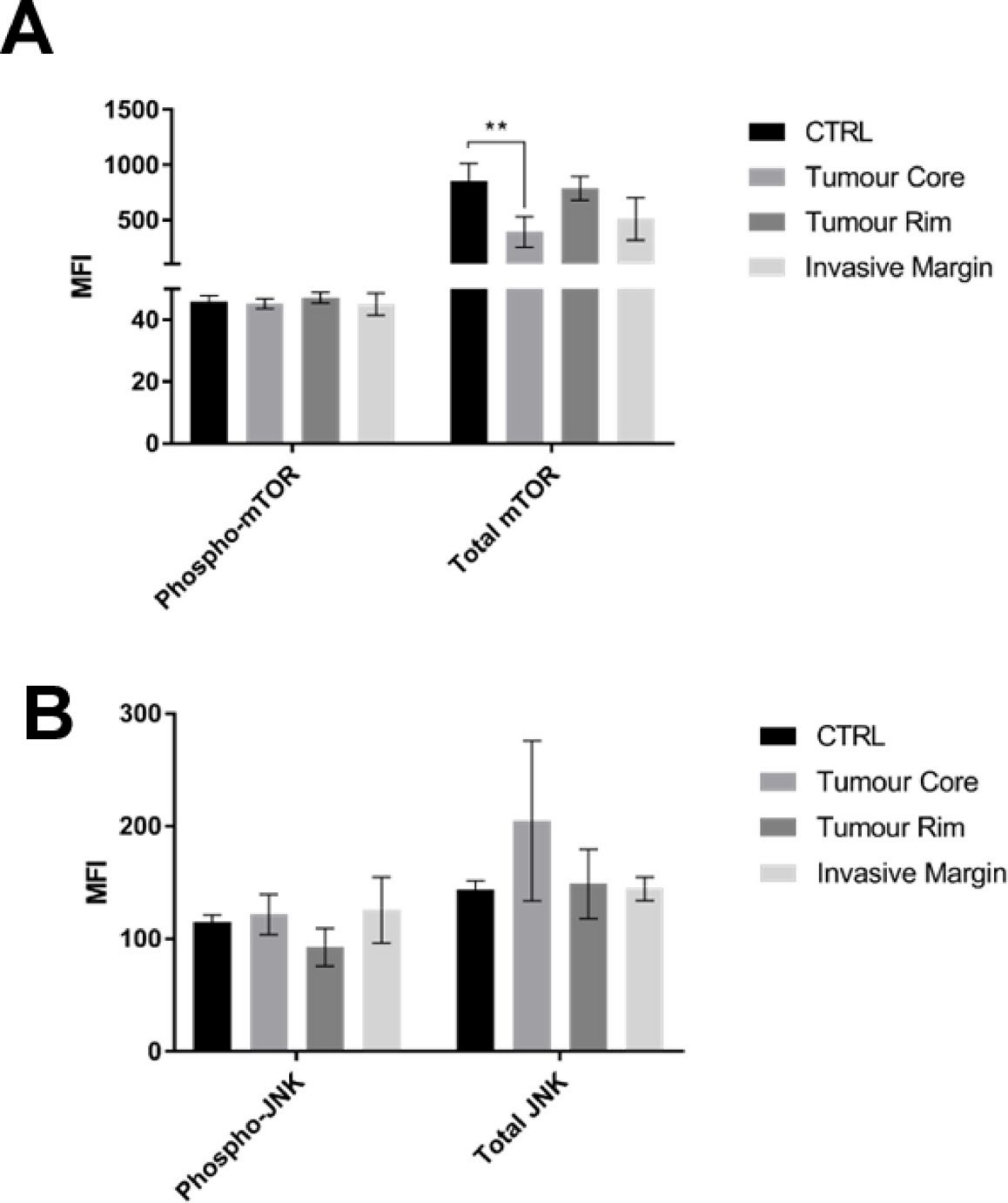
Protein phosphorylation profiling of mTOR and JNK. Multiple comparisons of median fluorescent intensity (MFI) from Milliplex (**A**) phospho-mTOR, total mTOR, (**B**) phospho-JNK and total JNK analysis, between intra-tumour regions and prefrontal cortex control (CTRL). Data was analysed by non-parametric one-way ANOVA (Kruskal–Wallis test) and statistical significance set at *P < 0.05; **P < 0.01. Mean ± SEM plotted. Multiple comparisons revealed significance for following regions: for total mTOR CTRL vs. Tumour Core: *p* = 0.0081.

We next conducted specific Milliplex^®^ assays to determine mTOR and JNK total protein and phosphorylated protein expression. No significant differences in phosphorylated mTOR and JNK proteins were observed between intra-tumour regions and non-diseased brain tissues, with only the tumour core exhibiting a significant downregulation of total mTOR protein (Fig. 2).

### 1D-polyacrylamide gel electrophoresis (ID-PAGE) profiling reveals a broadly comparable GBM and normal brain proteome

Electrophoretic separation of proteins serves as a simple but powerful method to observe proteomic changes in tissues and can be used as a first step in high-throughput separation of complex protein mixtures. To explore changes more broadly in the GBM proteome, biopsy samples were fractionated using differential centrifugation to separate cytosolic and nuclear cellular compartments. Proteins from the crude cytosolic and nuclear fractions were separated by their denatured molecular weight using 1D-PAGE and compared to prefrontal cortex non-diseased controls. Colloidal blue staining allowed for visualisation of total protein to infer major proteomic differences in tumour vs. normal brain.

**Fig. 3:**
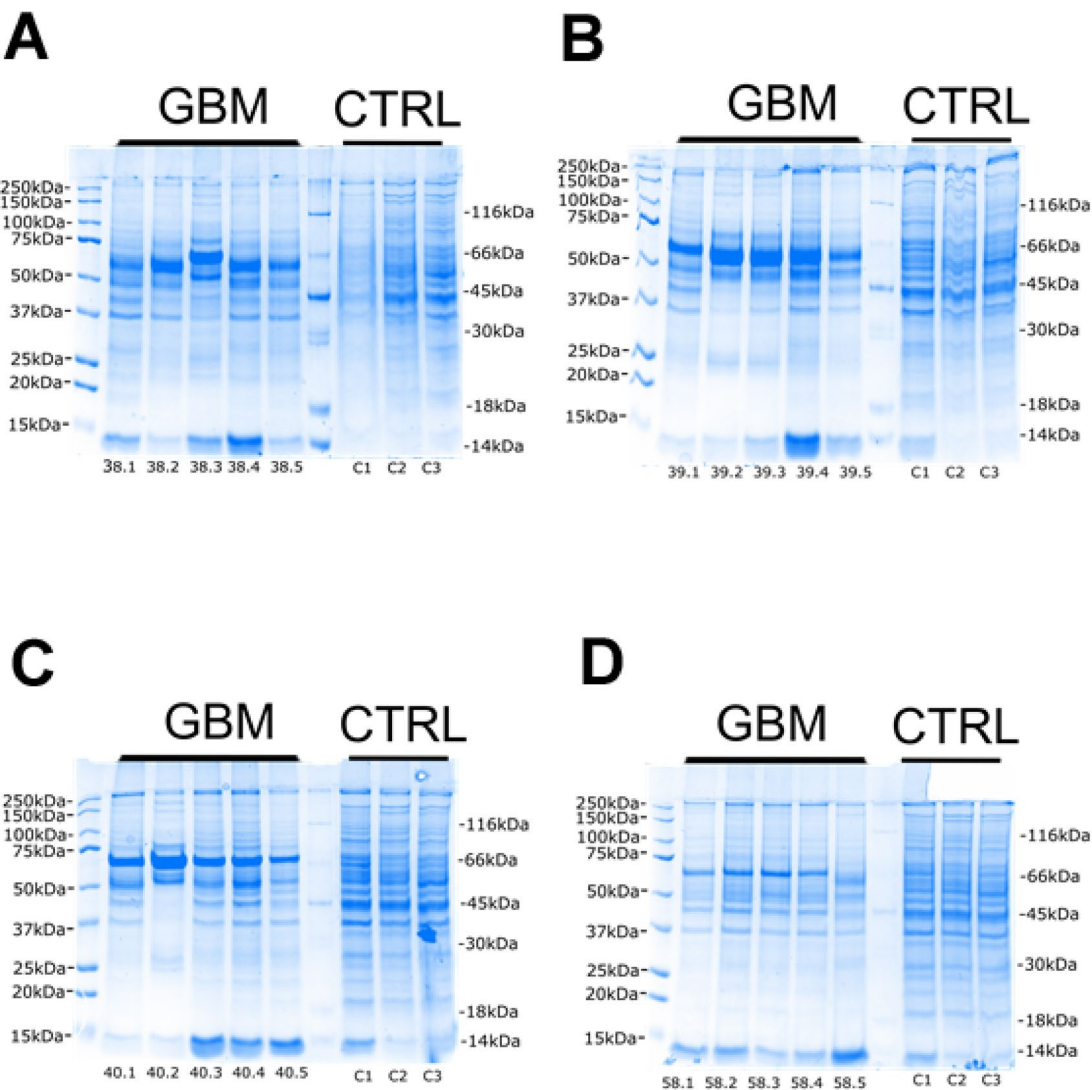
Gels of cytosolic proteins separated by 1D-PAGE and stained with colloidal blue for total protein visualisation. 25 µg of cytosolic proteins has been resolved for each sample from five intra-tumour regions of GBM tissue from patient 38 (**A**), 39 (**B**), 40 (**C**) and 58 (**D**), in addition to three control samples from non-diseased prefrontal cortex tissue. Lane 1 of each gel has been loaded with Pre-stained Protein Standard and Lane 7 loaded with Peppermint Stick Standard for phosphorylation specific dyes.

Resolution of cytosolic proteins displayed a broadly similar proteomic pattern in GBM tissue across four patients and five intra-tumour regions, relative to non-diseased brains (Fig. 3). Controls remained relatively constant in total protein staining across all gels suggesting that changes in proteomic content are due to distinct differences in samples rather than variations in gel loading and gel running. Total protein separation did reveal minimal levels of certain protein expression between 15 and 20 kDa, such as that on samples 38.2, 38.3 (Fig. 3A), 39.1 to 39.3 (Fig. 3B), 40.1, 40.2 (Fig. 3C) and 58.1 to 58.5 (Fig. 3D). Additionally, there was a visible increase in protein content at approximately 66 kDa for GBM samples 38 (Fig. 3A), 39 (Fig. 3B) and 40 (Fig. 3C) indicative of serum albumin which was confirmed by mass spectrometry (results not included).

**Fig. 4:**
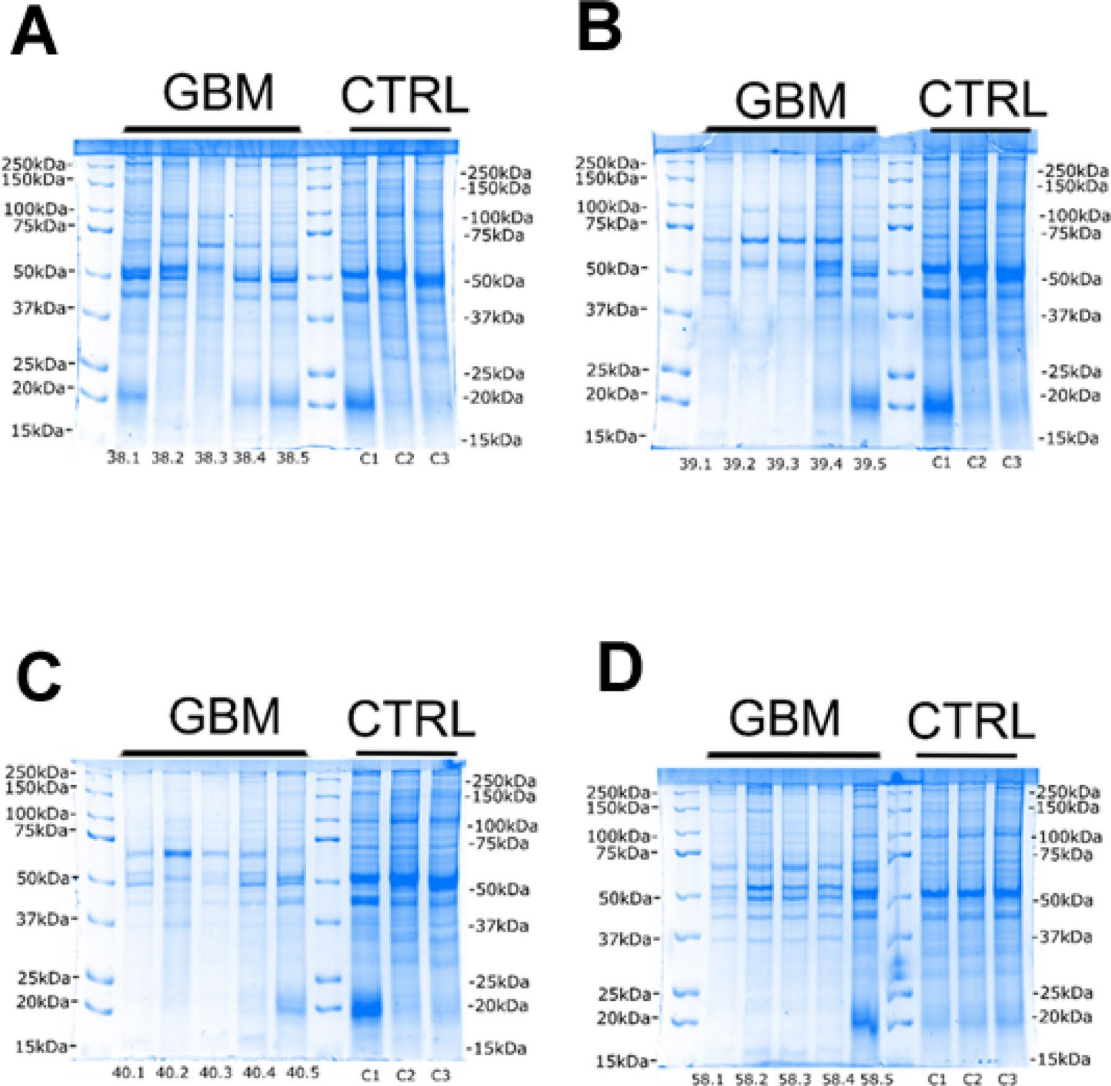
Gels of nuclear proteins separated by 1D-PAGE and stained with colloidal blue for total protein visualisation. 25 µg of nuclear proteins has been resolved for each sample from five different regions of GBM tumour tissue from four patients along with three control samples from healthy brain tissue (**A**–**D**). First and seventh lane of each gel has been loaded with Pre-stained Protein Standard for determination of denatured molecular weight.

Following cytosolic protein separations, we resolved proteins from nuclear fractions from GBM and non-diseased brain tissues (Fig. 4). There was a relative reduction of some protein bands in samples 39.1, 39.2 and 39.3 (Fig. 4B), samples 40.1, 40.2 and 40.3 (Fig. 4C) and sample 58.1 (Fig. 4D), particularly below a molecular weight of 37 kDa. Furthermore, there was relatively high expression of a protein band at approximately 50 kDa visible in samples 38.1, 38.2, 38.4 and 38.5 (Fig. 4A), samples 39.1, 39.4 and 39.5 (Fig. 4B), samples 40.1, 40.4, 40.5 (Fig. 4C), and all intra-tumour regions of sample 58 (Fig. 4D). Apart from these variations, the nuclear fraction displayed comparable total protein expression in GBM tissues relative to non-diseased brain tissues.

### 2D-PAGE detection of unique protein spots within GBM invasive margin

To further explore the feasibility of proteomic investigations, 2D-PAGE was used to resolve proteins according to their net charge (isoelectric point) and molecular weight. The separation of a complex protein mixture into its components through 2D-PAGE remains a powerful proteomic tool and often precedes sequencing methods for high-throughput analysis. The addition of a second dimension to molecular weight separation provides a robust method for the investigation of proteomic changes and can facilitate the resolution of protein post-translational modifications (PTMs) within complex samples. Protein PTMs often confer a difference in charge resulting in a change of a given protein’s isoelectric point (pI) and gel separation.

**Fig. 5:**
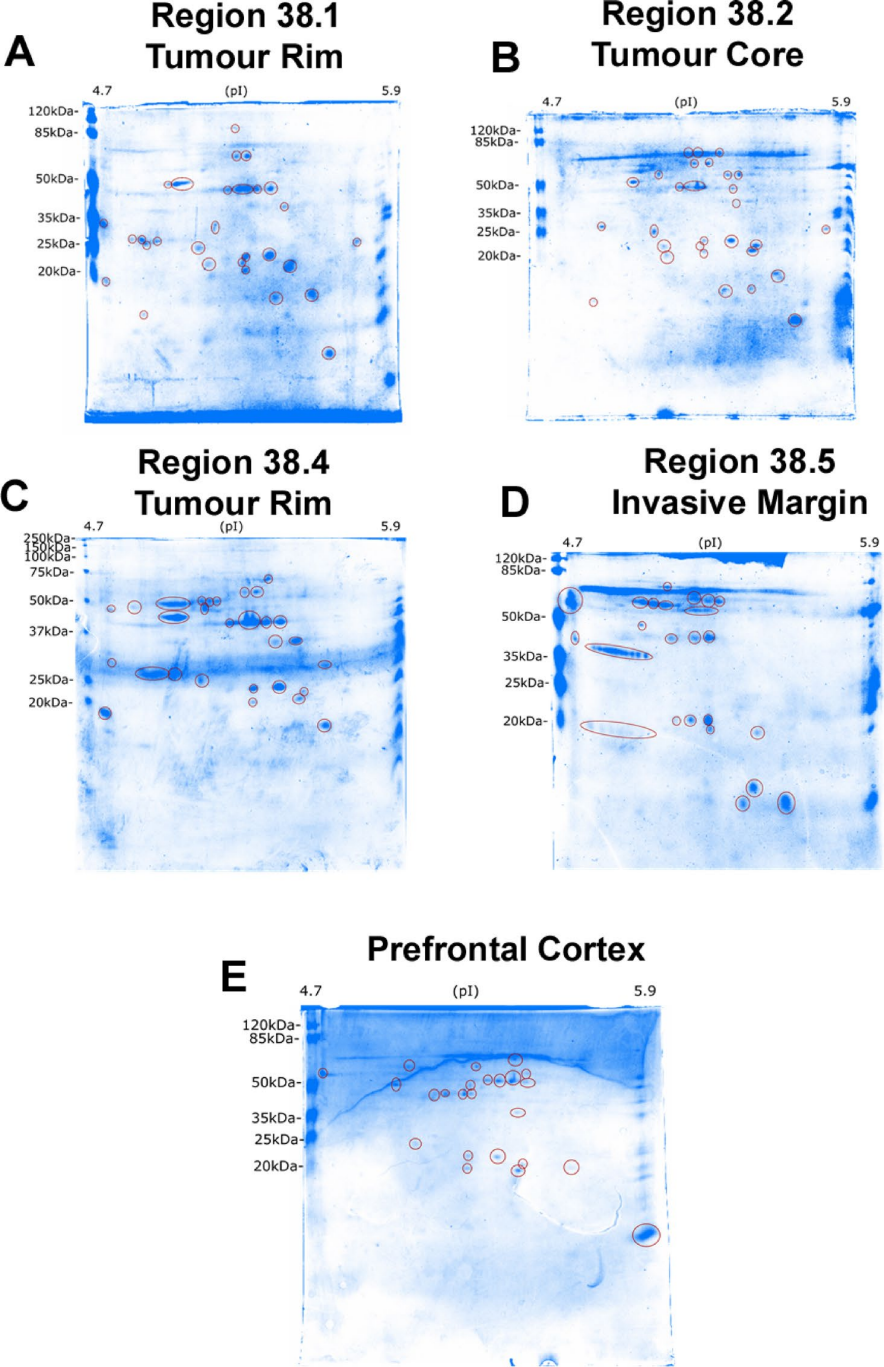
2D-PAGE resolution of four intra-tumour GBM regions and one non-diseased brain control. Cytosolic samples have been separated according to their isoelectric point (horizontal, pI 4.7 to 5.9 left to right) and molecular weight (vertical). Isoelectric focusing was performed on 17 cm immobilized pH gradient strips (IPG) of pH range between 4.7 and 5.9. IPG strips were then resolved on large 20 cm × 18 cm polyacrylamide gels for approximately 20 h and stained with colloidal blue overnight. (**A**–**D**) GBM intra-tumour regions from patient 38 as an exemplar. (**E**) Non-diseased human prefrontal cortex control. Each separate protein resolved by gel electrophoresis is marked (red circle).

Using patient 38 as an exemplar, GBM cytosolic proteome expression was compared between three intra-tumour regions (core, peripheral rim (two distinct biopsy fragments), and invasive margin), to that from the non-diseased prefrontal cortex. 2D-PAGE separation was successful at resolving the complex cytosolic protein lysates by pI and denatured molecular weight. To visualise the separation of proteins in each sample, colloidal blue protein staining was used. Many unique protein spots were identified for each intra-tumour region. Of particular interest were the series of spots at ~ 20 kDa and ~ 35 kDa between ~ 5.0 and 5.5 pH which are visible in the GBM invasive margin, but not detected in the tumour core, peripheral rim, or prefrontal cortex (Fig. 5).

**Fig. 6:**
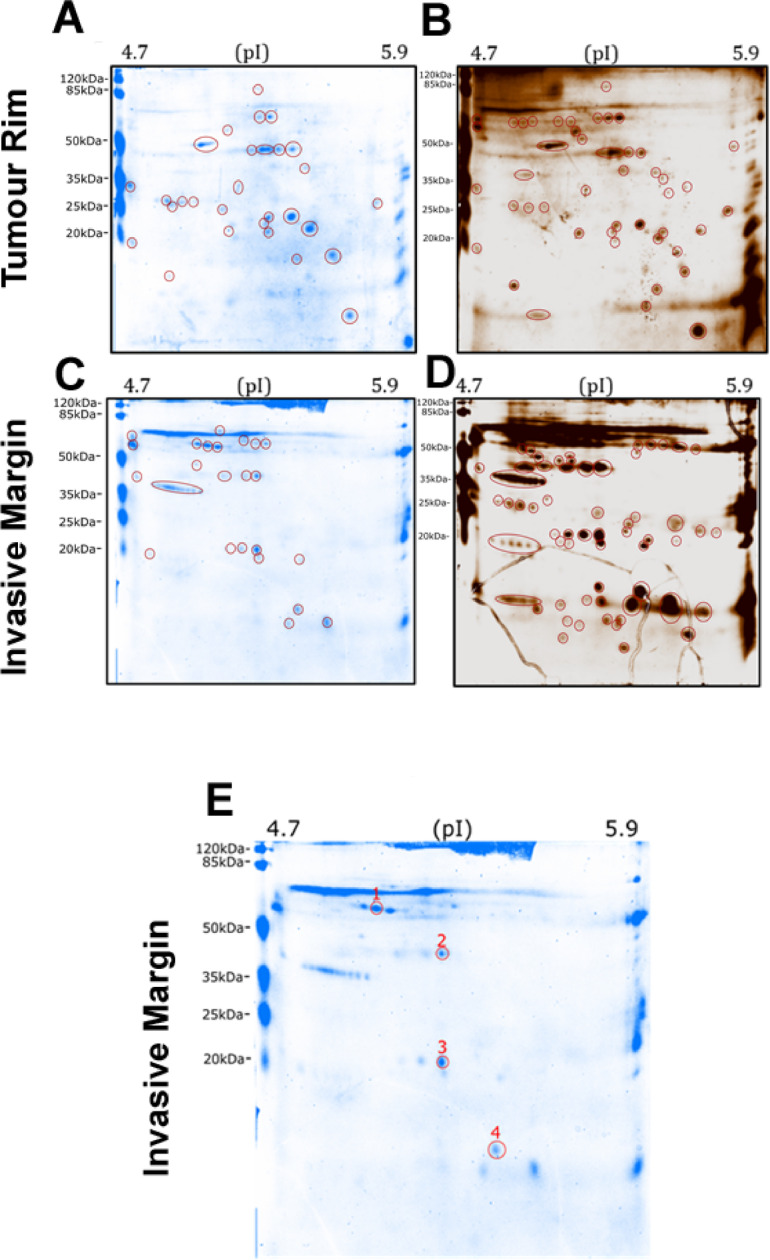
Comparison of two 2D-PAGE gels stained with colloidal blue followed by silver stain. (**A**–**D**) Representative GBM intra-tumour regions (tumour rim and invasive margin) from patient 38 resolved by 2D-PAGE to investigate both proteomic changes (colloidal blue) and possible post-translational modification (silver stain). Gels have been stained with colloidal blue stain to visualise total protein, de-stained with ultra-pure and re-stained with silver stain. Protein pI is represented horizontally from low pI (4.7) to high pI (5.9). Protein separation and detected proteins and possible post-translational modification have been marked with red circles on all images. (**A**) Twenty-nine proteins were resolved with colloidal blue stain, and (**B**) 45 distinct spots resolved by silver stain, for tumour rim. (**C**) Twenty-four proteins were resolved with colloidal blue stain, and (**D**) 60 distinct spots resolved by silver stain, for tumour invasive margin. Spots marked with elliptical red circle most likely represent a repeated pattern of a single protein and are marked as such. (**E**) Images of excised invasive margin proteins subjected to tandem mass spectrometry (MS/MS) analysis (red circles).

After destaining with ultrapure water, silver staining was performed, a more sensitive method which can detect as low as 0.25 ng of protein. Detection of the resolved proteins was greatly improved such that 45 distinct spots were detected in the patient 38 tumour peripheral rim (Fig. 6A,B) and 60 spots were identified in patient 38 invasive margin (Fig. 6C,D). There were also visible changes in protein expression across these intra-tumour regions, some of which may relate to protein PTMs, since PTMs are often observed as neighbouring spots with similar molecular weight and minor changes in pI. The regions that showed repeated patterns across varying pI may therefore represent the same protein (with different PTMs) and these are marked with horizontally elongated red circles (Fig. 6).

### Tandem mass spectrometry identifies proteins putatively associated with the GBM infiltrative tumour margin

**Fig. 7:**
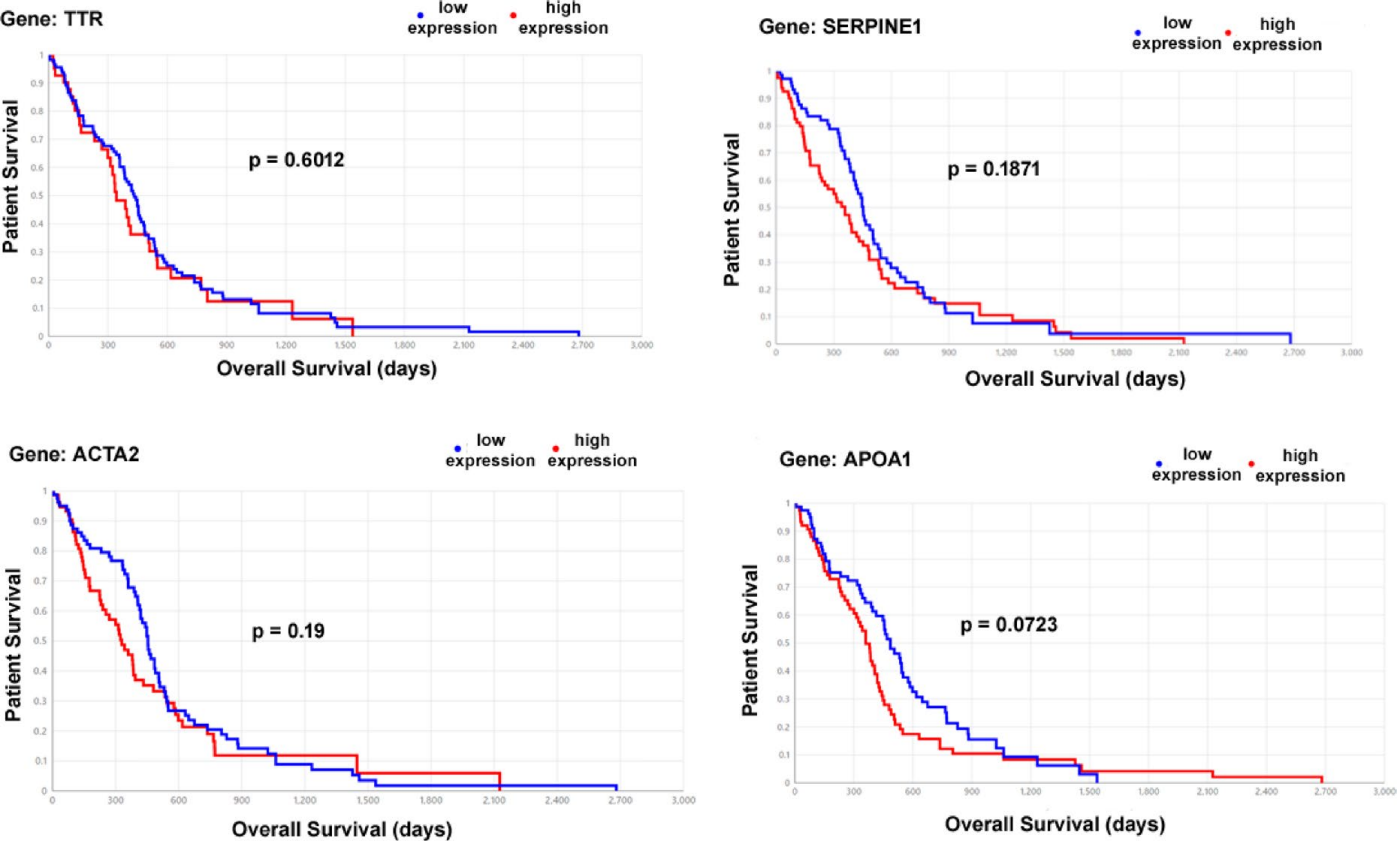
Kaplan Meier analyses from The Cancer Genome Atlas GBM RNAseq dataset. Genes (TTR, SERPINE1, ACTA2, APOA1) encoding transthyretin, α1-antitrypsin, actin and apolipoprotein A1 respectively, reveal no significant association with patient overall survival, with APOA1 trending toward significance (*p* = 0.0723).

With an emphasis on identifying specific protein spots in the clinically-relevant invasive margin samples from patient 38, we conducted tandem mass spectrometry (MS/MS), an analytical method often used as a follow-up to electrophoretic separation which enables the identification of unknown polypeptide sequences in specific bands or spots. Identified amino acid sequences can then be matched with available protein databases such as Swiss-Prot and UniProt. Four invasive margin proteins were chosen and excised from 2D-PAGE gels, prior to MS/MS analysis (Fig. 6E), and these were identified as cytoplasmic proteins α-trypsin, actin, apolipoprotein A1 and transthyretin which may putatively be associated with a GBM infiltrative phenotype (Table 3). To determine whether these four candidate invasive margin proteins could be stratified to patient survival, Kaplan Meier analyses was conducted using The Cancer Genome Atlas (TCGA) GBM RNAseq dataset for genes (TTR, SERPINE1, ACTA2, APOA1) encoding transthyretin, α1-antitrypsin, actin and apolipoprotein A1 respectively. No significant association with patient overall survival was evident, with only APOA1 trending toward significance (*p* = 0.0723) (Fig. 7).

**Table 3:** MS/MS identified proteins showing MASCOT protein score, molecular mass of protein in Daltons (Da), and proteins matched on the SwissProt database.

2D-PAGE			
Spot no	Score	Mass	Protein match (SwissProt)
1	467	46,878	Alpha-1-antitrypsin OS
2	108	42,052	Actin, cytoplasmic 1 OS
3	269	30,759	Apolipoprotein A-I OS
4	106	15,991	Transthyretin OS

Organism Species (OS)—human.

Overall, this data suggests that 2D-PAGE with silver staining has been successful at resolving distinct proteomic changes between tumour sites and should provide the basis for further exploration of the proteome at these different tumour regions.

## Discussion

Various protein changes and modifications were observed in separate tumours and across different tumour regions from the same tissue providing evidence for inter- and intra-tumour heterogeneity in the GBM samples. Milliplex^®^ multiplex assays revealed significant differences in the expression of key signalling kinases and transcription factors including p38, Akt, CREB and STAT3/5. The upregulation of CREB and Akt across the GBM invasive margin of all four tumours studied, relative to non-diseased brain tissues, is suggestive of a role for this transcriptional factor and protein kinase in tumour infiltration. Recently, glioblastoma stem cell invasion in an orthotopic xenograft model was promoted via an Orphan Receptor 1/HER2-CREB signalling axis^17^, and an ABCF1-CXCL12-CXCR4 axis was recently shown to promote GBM invasion by activating the PI3K-Akt signalling pathway^18^. As these studies utilised GBM experimental models, our observation of significantly upregulated CREB and Akt expression in the 5ALA-derived invasive margin, offers complementary clinical relevance and encourages further exploration of the functional association of CREB and Akt pathways in the transition from residual disease infiltration to the emergence of GBM recurrence within a therapeutic intervention context. Upregulated STAT3 expression in the peripheral rim and invasive margin (but not tumour core) relative to non-diseased brain tissues, may hint at STAT3-associated pro-invasive and immune microenvironment modulatory effects as reported across pan-cancer studies^19,20^. Whilst we demonstrated the feasibility of applying 1D-PAGE to intra-tumour GBM samples, this method yielded only minor differences when compared to non-diseased brain tissues. Results showed a general absence of certain lower molecular weight proteins of cytosolic and nuclear fractions, indicative of differential protein expression. We also compared the detection of two total protein stains on large (20 cm) 2D-PAGE gels. We used silver stain and colloidal blue which vary in sensitivity with silver stain being approximately 30-fold more sensitive and this detected approximately twice as many proteins than colloidal blue in both gels. We also observed changes in specific areas of 2D gels that may correspond to the PTM of proteins. Hence, although ‘bottom-up’ shotgun peptide-based MS approaches have superseded 2D-PAGE methods, the latter method facilitates detection of proteoforms and unequivocal protein identification that comes from ‘top-down’ spot-picking prior to mass analysis^21^. Therefore, further analysis of these regions by mass spectrometry will be needed to identify the specific proteins and their PTMs. Our mass spectrometry analyses of cytoplasmic proteins yielded the identification of α-trypsin, actin, apolipoprotein A1 and transthyretin from excised 2D-PAGE protein bands; proteins which may putatively be associated with a GBM infiltrative phenotype. It is intriguing to note that α-trypsin, apolipoprotein A1 and transthyretin have not been reported previously in the context of GBM invasion. Nevertheless, α-1 antitrypsin has been shown to promote lung cancer invasion, whereby α-1 antitrypsin functions as a critical effector of mutant p53^22,23^, apolipoprotein A1 is an independent prognostic marker for invasive ductal carcinoma likely attributable to apolipoprotein A1 protein, promoting cholesterol release from cells to induce an anti-inflammatory and anti-apoptotic effect^24^, and transthyretin reported to promote hepatocellular cholangiocarcinoma invasion through activation of the NF-κB, p-ERK1/2, and p-AKT pathways^25^.

Although genes (TTR, SERPINE1, ACTA2, APOA1) encoding transthyretin, α1-antitrypsin, actin and apolipoprotein A1, respectively, were not significantly associated with overall survival, we note that all TCGA GBM transcriptomic data represents samples derived from the proliferative core and not infiltrative margin. Although genes (TTR, SERPINE1, ACTA2, APOA1) encoding transthyretin, α1-antitrypsin, actin and apolipoprotein A1 respectively, were not significantly associated with overall survival, we note that all TCGA GBM transcriptomic data represents samples derived from the proliferative core and not infiltrative margin. Whilst the ‘Ivy Glioblastoma Atlas’ reports comprehensive RNA-seq data for histologically defined GBM regions (including ‘Leading Edge’ and ‘Infiltrative Tumour’), insufficient survival data for patients who also had TTR, SERPINE1, ACTA2 and APOA1 expression data, precludes survival analyses for these four candidate proteins. Nevertheless, the Ivy Glioblastoma Atlas could effectively be used as a discovery dataset to identify significantly upregulated ‘Leading Edge’ genes (relative to all other intra-tumour regions) as putative pro-invasive factors, offering candidates for spatial proteomic validation (e.g., DISCO-MS) using independent GBM histological sections^26^.

Though many discoveries have been achieved through genomic screening of GBM tumours, there is an increasing necessity to delineate GBM tumours in terms of their active proteome, not least to enable rationale screening of putative therapy targets. As the post-genomic era has thus far failed to yield any molecular targeted therapies which have satisfied phase III clinical trial criteria, GBM research is required to fully appreciate intra-tumour genetic and molecular heterogeneity and focus on the next-generation of therapy targets predicated on the infiltrative tumour margin as a best indicator of residual disease. We have shown the feasibility of complementary protein profiling methods which can be used to identify putative protein signals in this region which represent candidate therapy targets to be explored further. We must note that in the absence of tumour purification (e.g., via 5ALA-based fluorescence-activated cell sorting) from tissue biopsies derived from the GBM invasive margin, as previously demonstrated by us for transcriptomic studies^15,16^, we cannot exclude that α-trypsin, actin, apolipoprotein A1 and transthyretin expression represents non-disease astrocytic or myeloid cell populations. Our study reveals the challenge in interrogating protein expression within the clinically relevant infiltrative margin of GBM, whereby tumour signal is confounded by non-disease neural and immune cells. Thereby, our findings highlight the critical need to analyse this tumour region using high resolution spatial omics methods such as ‘Three-dimensional imaging of solvent-cleared organs profiled by mass spectrometry’ (DISCO-MS). This may be coupled to 5-ALA neuro-navigation (to obtain deeply infiltrative GBM tissue for sectioning) and co-registered with histological staining^27^. Nevertheless, significantly higher expression of these molecular factors relative to GBM core regions, suggests these may be functionally associated with the GBM infiltrative margin.

## Methods

### Clinical samples

Proteomic characterization was performed on GBM tissue from brain tumour resections of patients undergoing 5-aminolevulinic acid (5ALA)-guided neurosurgery at Queen’s Medical Centre, University of Nottingham, UK (see Table 1 for patient demographics, tumour site and intra-tumour region descriptors). The intra-tumour regions and infiltrative margins were defined by fluorescence of tumour tissue after 5-ALA treatment and five intra-tumour regions were isolated per patient, including tumour core, peripheral (non-infiltrative) rim, and infiltrative margin. Samples were stored at − 80 °C until required. Non-diseased brain tissue samples were from the prefrontal cortex, Broadman’s area 9, and were dissected and stored at − 80 °C at the time of autopsy until required.

### Approval for human experiments

Ethical approval was obtained from the National Health Service Research and Ethics Committee East Midlands (Ref: 11/EM/0076), for the informed consented collection of brain scans, tumour tissue, storage, and molecular analyses, and were used in accordance with the Human Tissue Act (2004) and subjected to audits by the Human Tissue Authority. Brain tissue used as controls was from subjects who had died of natural causes or accidents and collected *post-mortem* with prior informed consent from the donors^28^. This brain tissue was sex and approximately age-matched with the GBM tissue (males aged 43, 66 and 78, and a female aged 57). The control (non-diseased) brain tissue was handled in accordance with the Human Tissue Act (2004) (UK) and was supplied by the Neuropsychopharmacology Research Group from the Department of Pharmacology of the University of the Basque Country (UPV/EHU) (https://www.ehu.eus/en/web/neuropsicofarmacologia/home). The brain tissue collection and processing were conducted in compliance with the research policies and ethical review boards for *post-mortem* brain studies (Basque Institute of Legal Medicine, Bilbao, Spain) and registered in the National Biobank Register of the Spanish Health Department with the number C.0000035 (https://biobancos.isciii.es/ListadoColecciones.aspx). The control brain tissue samples were from Brodman’s area 9 (dorsolateral prefrontal cortex (PFC), macroscopically dissected at autopsy^26^. This brain region contains a broad mixture of neuronal subtypes^29^ and glia, and provides a match for an anatomical subsite with a high incidence of GBM tumours^30^.

### Protein extraction and quantification

Brain tumour samples (approximately 50 to 60 mg), while still frozen, were cut into smaller fragments using a surgical scalpel, weighed, and placed in a glass Dounce homogeniser kept on ice and homogenised in 2 mL of homogenisation buffer (150 mM NaCl, 50 mM Tris–HCL pH 7.5, 1 mM EDTA, 1% (v/v) DTT, 1% (v/v) phosphatase inhibitor cocktail and 1 protease inhibitor cocktail tablet (Roche) using 20 even and slow strokes with the hand-held homogenizer. For fractionation, the homogenate was first centrifuged for 10 min at 500 × g at 4 °C to pellet the nuclear fraction and cell debris, which was re-suspended in 500 µL of homogenisation buffer. The supernatant from this spin was transferred into a new Eppendorf and spun at 21,300 × g for 40 min at 4 °C. The upper part of the sample was retained as a crude cytosolic fraction and the pellet was retained as a membrane-associated fraction. To determine the total protein concentration of cytosolic, membrane and nuclear fractionates from brain tissue homogenates, a modified Lowry assay^31^ was performed using a Bio-Rad *DC* kit. The unknown concentration of each GBM and control brain samples were compared to a set of protein standards (1.25 to 20 µg) prepared from dilution of a bovine serum albumin (BSA) 2 mg/mL standard with 10 mM Tris/HCL pH 8 as diluent. Samples were incubated with the BioRad assay kit reagents and absorbance was measured using a Multiskan Spectrum Microplate Reader at 740 nm excitation. Absorbance (nm)/amount of standard protein (µg) was plotted with the linearity of the equation of R^2^ falling in the range between 96 and 99%. The total protein concentration of each sample was calculated from the intercept of the line from the standard curve.

### Milliplex^®^ MAP assay

Three different Milliplex MAP kits (ThermoFisher) were used, with details of the target proteins provided in Supplementary Table 2. Protein homogenates were diluted in Milliplex assay buffer at a 1:1 dilution. Twenty-five µg of protein was prepared per well for each sample (concentration of 1 µg/µL). A black, clear bottom 96-well plate was pre-wetted with 50 µl of assay buffer on a rotary shaker for 10 min at room temperature. The assay buffer was decanted and 25 µl of each sample was added per well. 1 × bead suspension (prepared in advance) was vortexed for 10 s and 12.5 µL was added to each well loaded with the sample, and plates were incubated overnight at 4 °C on a rotary shaker. Next, a handheld magnet was attached to the plate and beads settled for 120 s. The magnet was then removed, and the plate was washed with 100 µL of assay buffer per well. Then, 12.5 µL of detection antibody was added and the plate was incubated for 1 h on a rotary shaker at room temperature. After incubation, a handheld magnet was attached to the plate and after 120 s, the detection antibody was decanted. Then, 12.5 µL of Streptavidin–Phycoerythrin (SAPE) was added and left to incubate on a rotary shaker for 15 min. Without removing SAPE, 12.5 µL of amplification buffer was added and the plate was further incubated for 15 min on a rotary shaker. The handheld magnet was attached, and the SAPE/amplification buffer was decanted. Finally, 100 µL of assay buffer was added to each well and the plate was analysed using a Luminex MAGPIX (ThermoFisher) using the system’s pre-programmed protocols.

### Preparation of gels for 1D-PAGE

Protein fraction extracts were prepared to a final concentration of 1 µg/µL in 10 mM Tris–HCL buffer pH 7.5 with 10 × reducing agent and 4 × LDS buffer (ThermoFisher). Samples were vortexed and heated to 70 °C for 10 min in a block heater to denature the proteins and afterwards cooled on ice. Samples in the buffer were then pulsed on a centrifuge for 5 s at 5000 × g to collect evaporated material and were either separated by 1D-PAGE immediately or stored at − 20 °C.

Hand-cast gels were prepared with a Bio-Rad Mini-PROTEAN system for 1D-PAGE gels (10.0 × 8.0 cm) and PROTEAN II system for 2D-PAGE large gels (20.0 × 18.3 cm). 1D-PAGE was performed either on large 12% or small 10% polyacrylamide gels overlaid with a 4% and 5% resolving gel, respectively. Small gels were prepared with 1.0 mm spacers and 10 well combs for 1D-PAGE and 17 cm IPG (immobilized pH gradient)-well comb for 2D-PAGE gels. Hand-cast gels were either used on the same day or stored at 4 °C for up to one week.

### 1D and 2D-PAGE

For 1D-PAGE, gels were placed in an electrophoresis tank (X-Cell SureLock Mini Cell or Mini-PROTEAN Tetra) and secured with tank gaskets. Gel wells were then loaded with 5 µl of protein standard, 20–25 µL (approximately 1 µg/µL concentration) of protein sample and the remaining empty wells were loaded with 1 × loading buffer. Electrophoresis was set to 125 V constant voltage and ran for approximately 95 min or until the tracking dye reached the bottom of the gel cassette. After the run, the casing of the cassettes was removed, and gels were washed in 10% (v/v) acetic acid and 50% methanol (v/v) solution to fix the proteins prior to gel staining.

For 2D-PAGE, the range of pH used in IEF is critical as proteins will migrate across the pH gradient to the point where they carry no net charge. For this study, broad pH (pH 3–10) strips were used first to view the majority of the proteins on one gel and then a narrower range was used (pH 4.7–5.9) for better resolution of target proteins. For small gels, 100 to 300 µg of proteins were used and for large 2D-PAGE gels, protein content was scaled up to 1000 µg. To remove contaminants and salts that could interfere with isoelectric focusing, acetone precipitation was performed. Acetone precipitation is used for the purification of proteins and removal of contaminants for downstream applications such as 2D-PAGE and mass spectrometry. Precipitation was performed with 100% acetone cooled to − 20 °C. A four times volume of acetone was added to the samples and the contents were vortexed for 20 s. Samples were then incubated on ice for 60 min. After the incubation, samples were centrifuged at 14,000 × g for 10 min at 4 °C and the supernatant was aspirated. The precipitate was washed 3 times with acetone and centrifuged at 14,000 × g. After the third wash, the pellet was air dried for approximately 15 min and resuspended in solubilisation solution for 2D-PAGE^32^. Prior to sample re-solubilisation, 0.5% (v/v) of IPG carrier ampholytes matching the strip pH (3–10 or 4.7–5.9), 1% (v/v) of 200 mM tributylphosphine reducing agent and 12 µL of de-streak reagent was added to rehydration buffer stock solution to make 1 mL of working solution. As per our previous report^32^, after a 1-h incubation period solubilized proteins were placed in a rehydration tray and overlaid with IPG strips (pH 3–10 or 4.7–5.9) gel side down. Active rehydration was performed at low voltage (50 V) for 16 h using the Protean IEF cell internal protocol.

Strips were equilibrated to solubilise focused proteins and allow SDS binding in preparation for the second dimension. DTT and iodoacetamide were used to reduce and then alkylate sulfhydryl groups, respectively. Then, 10 µL of protein standard was pipetted into the first well and the IPG strip was overlaid with warm molten 1% agarose to fix it in place. The 7 cm strips were resolved using Bio-Rad’s Mini Protean tetra-cell at a constant 125 V for 2 h. The 17 cm strips were resolved using Bio-Rad’s Protean II xi cell system with cold water cooling at 4 °C for 20 h with a constant current of 22 mA.

### Colloidal blue and silver staining

Total protein content in 1D and 2D gels was visualised using a colloidal blue staining kit (ThermoFisher). Resolved and fixed gel proteins were washed in a colloidal blue fixing solution containing 20% (v/v) methanol, 20% (v/v) stainer A for 10 min on a rotary shaker at room temperature. Then, 5% (v/v) of stainer B was added and gels were stained overnight. The next day, gels were destained several times with ultrapure water and then imaged with a ChemiDoc MP Imaging System (BioRad).

A SilverQuest™ kit (ThermoFisher) was used in this study for protein silver staining. After electrophoresis, gels were washed in ultrapure water and incubated in a fixative solution overnight. Fixative was decanted, and gels were washed in a 30% ethanol solution for 10 min. Ethanol was discarded, and a sensitizing solution was added. Gels were incubated in a sensitizing solution for 10 min on a rotary shaker. Gels were then washed in 30% ethanol and then in ultrapure water consecutively for 10 min. Silver stain solution was added, and gels were incubated for 15 min. Gels were then washed briefly in ultrapure water and then a developing solution was added. After protein staining had reached the desired intensity (5 to 10 min after adding the developing solution) stopper solution was added. Gels were washed in ultrapure water and then imaged using a ChemiDoc MP Imaging System (BioRad).

### In-gel proteolysis

Polyacrylamide gel bands and spots detected by chemical staining, were excised, and subjected to a general in-gel proteolytic protocol^33^ using DTT as reducing agent, iodoacetamide for sulphydryl alkylation and Promega sequencing grade Trypsin.

### Liquid-chromatography mass-spectrometry and data analysis

Capillary scale liquid chromatography used self-packed columns (0.5 × 50 mm, 2.7um, 90 Å, superficially porous HALO^®^ C18) chromatographed with a binary gradient of 5–90% (v/v) acetonitrile in 0.1% (v/v) formic acid at a flowrate of 0.01 mL min^−1^ achieved using flow-splitting from 0.25 mL min^−1^ (Thermo Scientific; Ultimate 3000). Mass spectrometry used a Q Exactive™ Hybrid Quadrupole-Orbitrap™ instrument (Thermo Scientific) equipped with an electrospray ion-source and operating in positive-ion data-dependant (ddMS2 with 2^+^, 3^+^ and 4^+^ charge state filtering) product ion acquisition.

Protein identification used the msconvert data processing application (proteowizard.sourceforge.net) followed by an open search of the SwissProt database using the MASCOT MS/MS ions application^34^.

### Statistical analysis

Using GraphPad-Prism (ver. 7.03) software, ANOVA and Student’s t-test were used, with statistical significance accepted at a *p* < 0.05 threshold. For data not fitting a Gaussian distribution (non-parametric), a Mann–Whitney test was used. Error bars plotted on graphs are presented as standard error of the mean unless otherwise stated.

## Electronic supplementary material

Below is the link to the electronic supplementary material.

Supplementary Material 1

## Data Availability

All data presented in this study are available from the corresponding author upon reasonable request.
