# Supplementary material for: Regional profiling reveals a distinct glioblastoma infiltrative margin proteome
Source: Sci Rep. 2025 Jul 5;15:24021. doi: 10.1038/s41598-025-09228-z (PMC12228809; doi:10.1038/s41598-025-09228-z)
Supplement: Supplementary file 1 — Supplementary Material 1 [file 41598_2025_9228_MOESM1_ESM.docx]

|  | Cellular Fraction (conc. mg/mL) | | |
| --- | --- | --- | --- |
| Sample ID. |  | Cytosol | Nuclear |
| 38.1 |  | **3.461** | **7.992** |
| 38.2 |  | **3.733** | **8.831** |
| 38.3 |  | **3.880** | **8.176** |
| 38.4 |  | **3.572** | **8.031** |
| 38.5 |  | **3.641** | **7.865** |
|  |  |  |  |
| 39.1 |  | **4.053** | **6.696** |
| 39.2 |  | **4.020** | **6.433** |
| 39.3 |  | **4.363** | **6.606** |
| 39.4 |  | **4.562** | **7.357** |
| 39.5 |  | **3.840** | **7.248** |
|  |  |  |  |
| 40.1 |  | **3.846** | **6.654** |
| 40.2 |  | **3.809** | **7.519** |
| 40.3 |  | **3.869** | **6.834** |
| 40.4 |  | **4.054** | **7.198** |
| 40.5 |  | **3.313** | **8.160** |
|  |  |  |  |
| 58.1 |  | **4.978** | **5.666** |
| 58.2 |  | **5.673** | **5.892** |
| 58.3 |  | **5.381** | **5.258** |
| 58.4 |  | **5.565** | **5.904** |
| 58.5 |  | **5.958** | **7.019** |
|  |  |  |  |
| CTRL1 |  | **3.729** | **7.757** |
| CTRL2 |  | **3.799** | **7.596** |
| CTRL3 |  | **3.817** | **6.830** |
| CTRL4 |  | **3.852** | **7.021** |

**Supplementary Table 1: Protein quantification of each sample and their fractionated protein content**. Results obtained from a modified Lowry protein quantification. Experimental samples obtained from patients 38, 39, 40, and 58 correspond to intra-regions described in Table 1. CTRL; control samples from the prefrontal cortex.

| **Milliplex MAP Kit** | **Target Proteins** |
| --- | --- |
| Phospho/Total mTOR | Total mTOR, phospho-mTOR (Ser2448) |
| Phospho/Total JNK | Total JNK, phospho-JNK (Thr183/Tyr185) |
| 9-Plex Multi-Pathway  (48-680MAG) | ERK/MAP kinase (Thr185/Tyr187), Akt (Ser473), STAT3 (Ser727), JNK (Thr183/Tyr185), p70 S6 kinase (Thr412), NF-ĸB (Ser536), STAT5A/B (Tyr694/699) CREB (Ser133), p38 (Thr180/Tyr182) |

**Supplementary Table 2: Kits used in the Milliplex MAP assay with their corresponding target proteins and phosphorylation site**. JNK and mTOR kits were specific for total and phosphorylated protein expression. 9-Plex targeted a wide range of phosphorylated proteins. Specific site of phosphorylation is described in the table.
